# Addendum: Kotál et al. *Ixodes ricinus* Salivary Serpin Iripin-8 Inhibits the Intrinsic Pathway of Coagulation and Complement. *Int. J. Mol. Sci.* 2021, *22*, 9480

**DOI:** 10.3390/ijms222011271

**Published:** 2021-10-19

**Authors:** Jan Kotál, Stéphanie G. I. Polderdijk, Helena Langhansová, Monika Ederová, Larissa A. Martins, Zuzana Beránková, Adéla Chlastáková, Ondřej Hajdušek, Michail Kotsyfakis, James A. Huntington, Jindřich Chmelař

**Affiliations:** 1 Department of Medical Biology, Faculty of Science, University of South Bohemia in České Budějovice, Branišovská 1760c, 37005 České Budějovice, Czech Republic; jan.kotal@nih.gov (J.K.); hlanghansova@prf.jcu.cz (H.L.); ederom01@prf.jcu.cz (M.E.); beranz02@prf.jcu.cz (Z.B.); chlasa00@prf.jcu.cz (A.C.); kotsyfakis@paru.cas.cz (M.K.); 2 Laboratory of Genomics and Proteomics of Disease Vectors, Institute of Parasitology, Biology Center CAS, Branišovská 1160/31, 37005 České Budějovice, Czech Republic; larissa.martins@nih.gov; 3 Cambridge Institute for Medical Research, Department of Haematology, University of Cambridge, The Keith Peters Building, Hills Road, Cambridge CB2 0XY, UK; stephanie.polderdijk@cantab.net (S.G.I.P.); jah52@cam.ac.uk (J.A.H.); 4 Laboratory of Vector Immunology, Institute of Parasitology, Biology Center CAS, Branišovská 1160/31, 37005 České Budějovice, Czech Republic; hajdus@paru.cas.cz

The accession code will be added in Section 2.8. Structural Features of Iripin-8: The coordinates and structure factors of Iripin-8 are deposited in the Protein Data Bank under accession code 7PMU.

This addendum does not cause any changes to the results or conclusions in the original published paper [1].

## References

[1] Kotál J., Polderdijk S.G.I., Langhansová H., Ederová M., Martins L.A., Beránková Z., Chlastáková A., Hajdušek O., Kotsyfakis M., Huntington J.A. (2021). *Ixodes ricinus* Salivary Serpin Iripin-8 Inhibits the Intrinsic Pathway of Coagulation and Complement. Int. J. Mol. Sci..

