# Supplementary material for: Ixodes ricinus Salivary Serpin Iripin-8 Inhibits the Intrinsic Pathway of Coagulation and Complement
Source: Int J Mol Sci. 2021 Aug 31;22(17):9480. doi: 10.3390/ijms22179480 (PMC8431025; doi:10.3390/ijms22179480)
Supplement: Supplementary file 1 [file ijms-22-09480-s001.zip › ijms-1291351-supplementary.pdf]

Supplementary Materials

Ixodes ricinus Salivary Serpin Iripin-8 Inhibits the Intrinsic Pathway of Coagulation and Complement

Supplementary Table S1. List of primers.

Amplicon name	Forward primer 5´ - 3´	Reverse primer 5´ - 3´	Amplicon length
Iripin-8 RT-PCR	GACTCGGTTAATCCTCCTCAAC	ATGGGTACCTGGACCTTCT	123 bp
I.ricinus ef1 RT-PCR	CTGGGTGTGAAGCAGATGAT	GTAGGCAGACACTTCCTTCTG	105 bp
Iripin-8 cloning	CACAGAGAACAGATTGGTGGACAAGACGAAATCAGCCAAG	GTCTCCTGAGTTCTAGAGTACTTTATCAGAGGGCGTTGATCT	1207 bp
Iripin-8 RNAi	ACTACCTGGGGCTCAATCTT	CCTGTTGCTAACCCAGTGT	401 bp
Borrelia flagellin	AGCAAATTTAGGTGCTTTCCAA	GCAATCATTGCCATTGCAGA	173 bp
Mouse β-actin	AGAGGGAAATCGTGCGTGAC	CAATAGTGATGACCTGGCCGT	138 bp
Borrelia flagellin probe	TGCTACAACCTCATCTGTCATTGTAGCATCTTTTATTTG		
Mouse β-actin probe	CACTGCCGCATCCTCTTCCTCCC		

Supplementary Table S2. Data processing, refinement, and model.

**Crystal**		
Space Group	F222	
Cell dimensions (Å)	a = 96.95 b = 130.67 c = 151.09	
(°)	α = β = γ = 90	
Solvent content (%)	56.1	
**Data Processing Statistics**		
Wavelength (Å)	0.91587 (Diamond, beam line I04-1)	
Resolution (Å)	75.54-1.89	1.93-1.89
Total reflections	327544	22074
Unique reflections	38420	2465
Multiplicity	8.5	9.0
$<I/\sigma(I)>$	8.9	1.6
Completeness (%)	100.0	100.0
R_{merge}	0.124	1.441
CC(1/2)	0.998	0.594
**Model**		
Number of atoms modeled:		
Protein	2882	
PEG	65	
Water	78	
B-factors (Å^2)		
Average	41.0	
Protein	40.4	
Ions/ligand	67.8	
Water	42.1	
**Refinement statistics**	69.31-1.89Å	1.94-1.89Å
Reflections in working/free set	36491 / 1926	2677/ 136

R-factor/R-free (%)	20.40 / 24.34	44.0 / 49.1
r.m.s. deviation of bonds(Å)/angles (°) from ideality	0.010 / 1.568	
Ramachandran Favoured	97.89%	
Molprobity Score	1.38 (98th percentile)	

Phylogenetic analysis of Iripin-8 group among tick species

In order to show the conserved nature of Iripin-8-like serpins in ticks, an alignment was produced by using the ClustalW algorithm in BioEdit version 7.2.5 [1] (Supplementary Figure S1A) and an unrooted phylogenetic tree was built using MEGA X software [2] (Supplementary Figure S1B). Interestingly, the RCL and adjacent regions are 100% conserved, unlike the rest of the sequence that seems to undergo evolutionary changes, as evidenced by strongly supported species-specific branches of the phylogenetic tree (Supplementary Figure S1B).

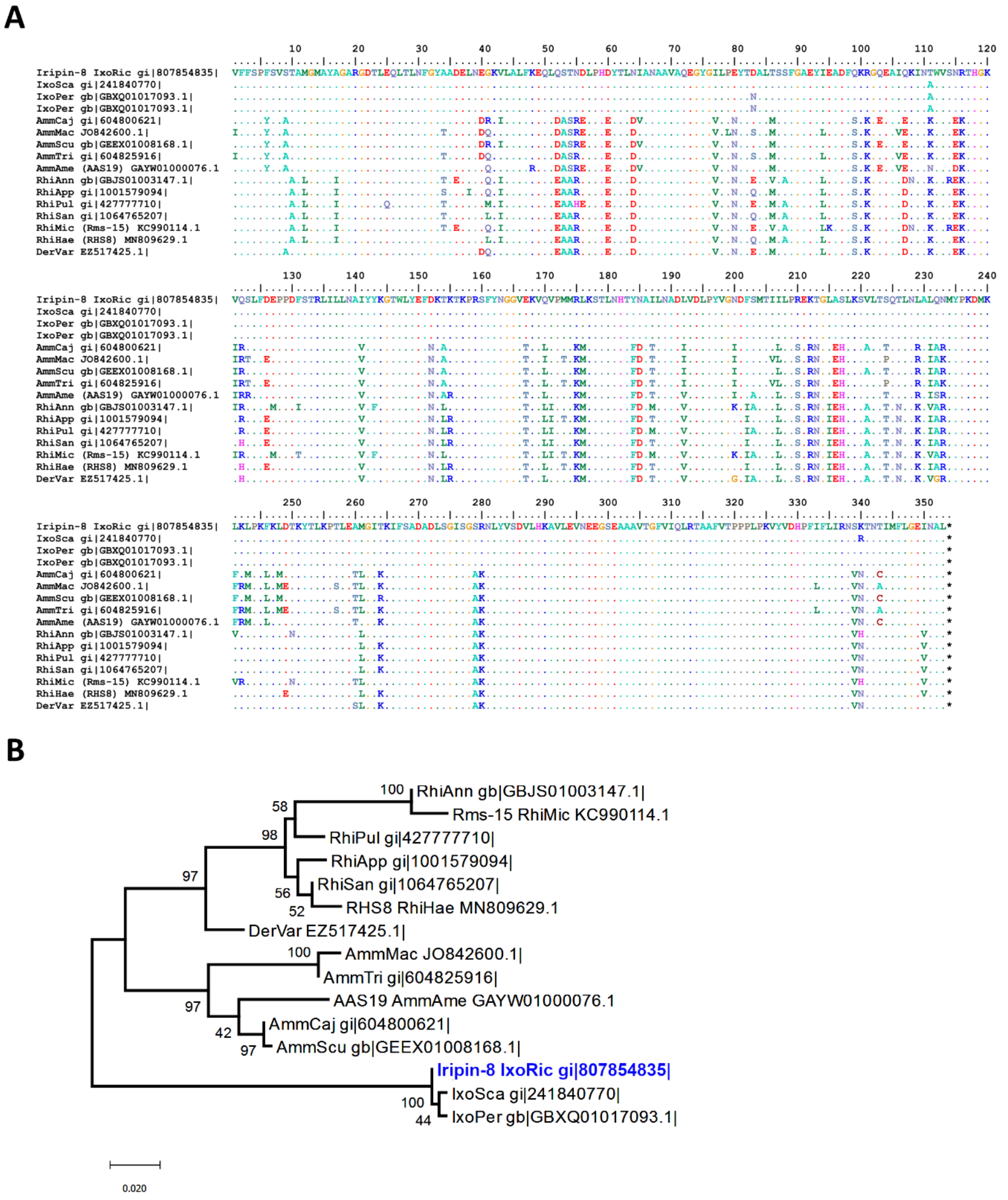

Supplementary Figure S1. Alignment and phylogenetic analysis of Iripin-8. (**A**) Alignment of members of the Iripin-8 group of tick serpins. The conserved area, including hinge region and RCL, is between residues 290-330. (**B**) Phylogenetic reconstruction of serpins from the Iripin-8 group. See Supplementary Methods for details of the analysis.

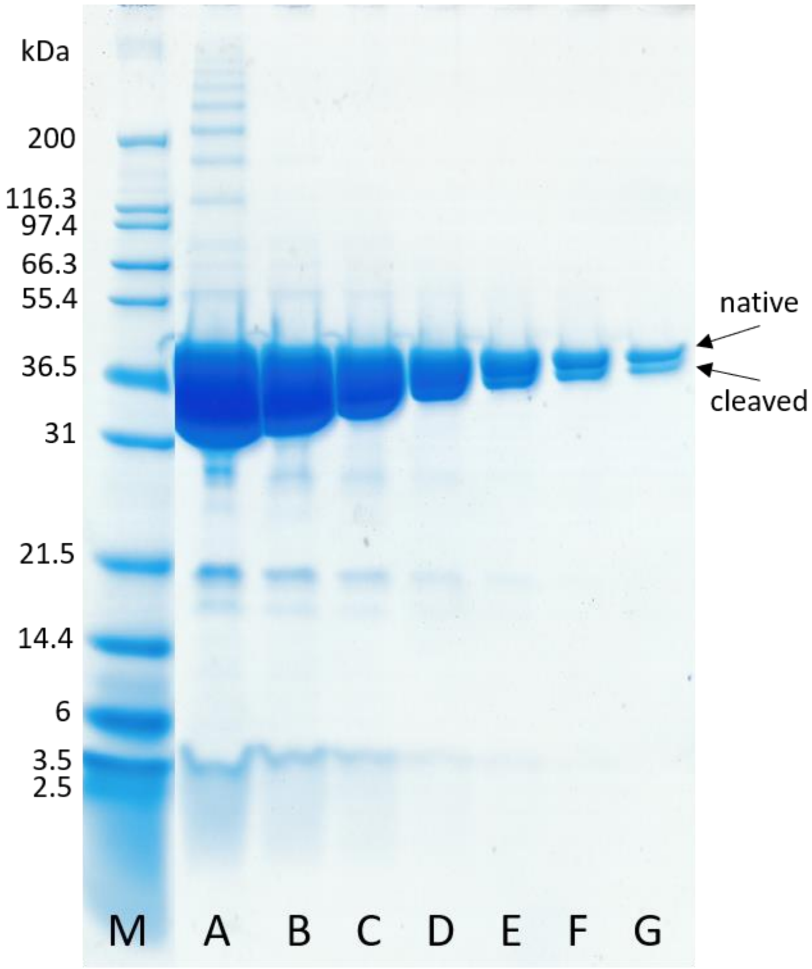

Supplementary Figure S2. Analysis of Iripin-8 purity by SDS-PAGE. Iripin-8 was analyzed by a reducing SDS-PAGE gel. M: Molecular weight marker, A-G: Iripin-8 with load of 50, 25, 12.5, 6.2, 3.1, 1.55, 0.8 mg per well. Arrows show Iripin-8 in its native and cleaved states.

Circular dichroism (CD) spectroscopy

In order to verify a correct fold of Iripin-8 recombinant protein, we performed CD spectroscopy analysis. Prior to analysis, buffer of Iripin-8 solution was exchanged for 20 mM NaH_2PO_4, 150mM NaF, pH 7.4. CD spectra were obtained using a JASCO J-810 spectropolarimeter at 22°C at wavelengths ranging from 190 to 300 nm using a 0.1 mm path-length cuvette. As shown in Supplementary Figure 2, Iripin-8 displays properties typical for the serpin secondary structure [3, 4].

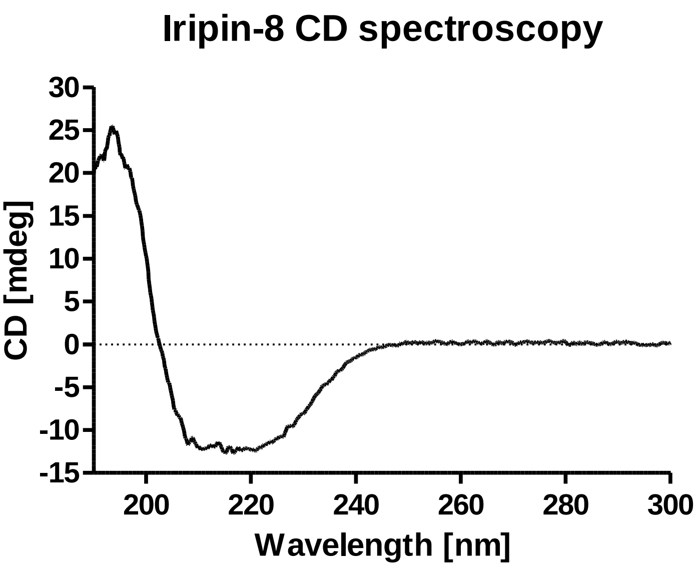

Supplementary Figure S3. CD spectrogram of Iripin-8. Iripin-8 shows a similar CD spectrum to other serpins due to their highly conserved structure.

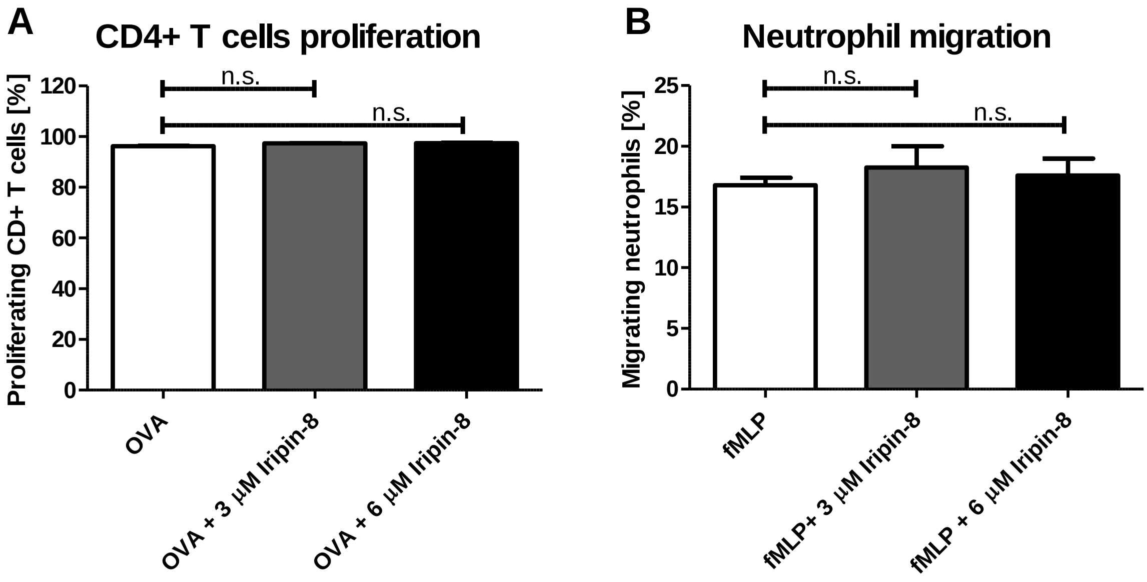

Supplementary Figure S4. Effect of Iripin-8 on T cell proliferation and neutrophil migration. (**A**) Splenocytes from OT-II mice were pre-incubated with Iripin-8 and stimulated by OVA peptide. Percentages of proliferating $CD4^+$ T cells were evaluated after 72 hours by flow cytometry. (**B**) Mouse primary bone marrow neutrophils were pre-incubated with Iripin-8 and subjected to migration towards fMLP in a Boyden chamber. Figure shows the percentage of neutrophils migrating from an insert with a 3 µm membrane to a compartment with fMLP. Both experiments were performed as three biological replicates.

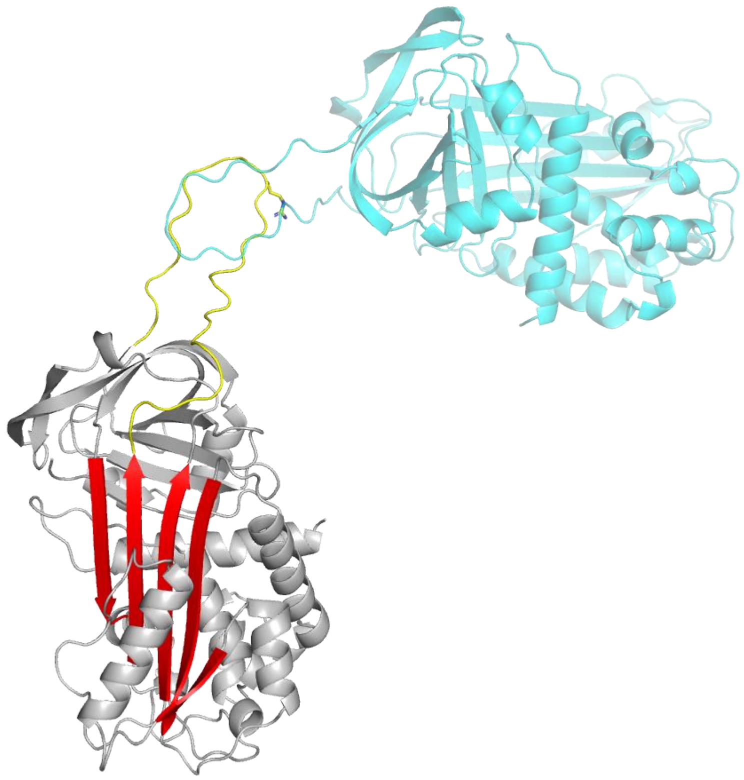

Supplementary Figure S5. A ribbon diagram of two Iripin-8 symmetry-related molecules in a crystal contact via their extended rigid RCL. RCL of one Iripin-8 molecule is depicted in yellow, while the second Iripin-8 molecule is all blue.

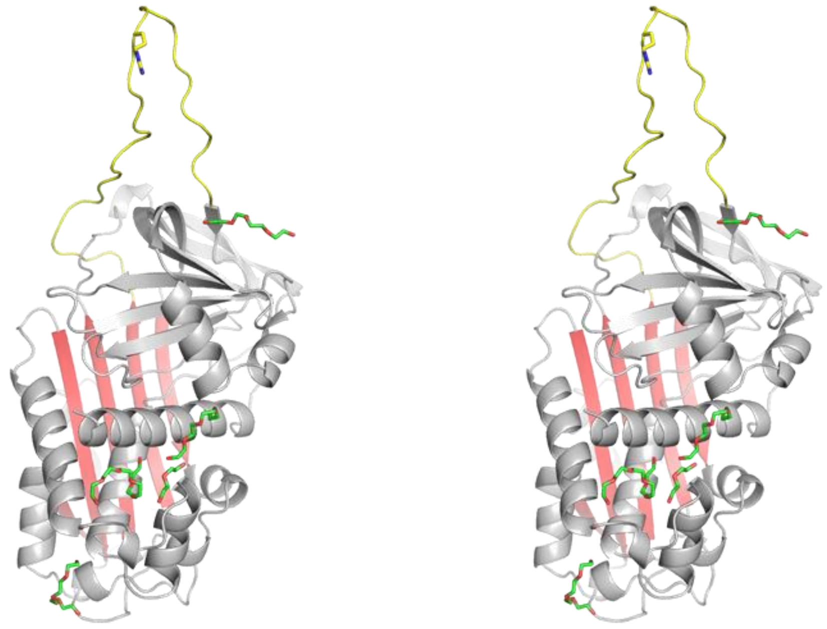

Supplementary Figure S6. Stereo view of a ribbon diagram of Iripin-8 with highlighted (green) molecules of PEG bound to the structure including a deep cavity in the core structure between helices A, B, and C.

Supplementary Methods

Evolutionary analysis by the maximum likelihood method

The evolutionary history was inferred using the maximum likelihood method and Tamura-Nei model [5]. The tree with the highest log likelihood (-1062.81) is shown. The percentage of trees out of 1000 replications by bootstrap method in which the associated taxa clustered together is shown next to the branches. The tree is drawn to scale, with branch lengths measured in the number of substitutions per site. Initial tree(s) for the heuristic search were obtained automatically by applying Neighbor-Join and BioNJ algorithms to a matrix of pairwise distances estimated using the Tamura-Nei model and then selecting the topology with superior log-likelihood value. There were 355 positions in the final dataset. Evolutionary analyses were conducted in MEGA X [2].

References

1. Hall, T. A., BioEdit : a user-friendly biological sequence alignment editor and analysis program for Windows 95/98/NT. *Nucleic Acids Symp. Ser.* **1999,** 41, 95-98.
2. Kumar, S.; Stecher, G.; Li, M.; Knyaz, C.; Tamura, K., MEGA X: Molecular Evolutionary Genetics Analysis across Computing Platforms. *Mol Biol Evol* **2018,** 35, (6), 1547-1549.
3. Akazawa, T.; Ogawa, M.; Hayakawa, S.; Hirata, M.; Niwa, T., Structural change of ovalbumin-related protein X by alkali treatment. *Poult. Sci.* **2018,** 97, (5), 1730-1737.
4. Yang, L.; Irving, J. A.; Dai, W.; Aguilar, M. I.; Bottomley, S. P., Probing the folding pathway of a consensus serpin using single tryptophan mutants. *Sci. Rep.* **2018,** 8, (1), 2121.
5. Tamura, K.; Nei, M., Estimation of the number of nucleotide substitutions in the control region of mitochondrial DNA in humans and chimpanzees. *Mol Biol Evol* **1993,** 10, (3), 512-26.
